# The consolidation of surgery for hypertrophic obstructive cardiomyopathy in Asia and the Pacific Rim

**DOI:** 10.1177/02184923211057323

**Published:** 2021-11-06

**Authors:** Eduard Quintana, Carlos A Mestres

**Affiliations:** 1 Department of Cardiovascular Surgery, Hospital Clinic, 16493 University of Barcelona, Barcelona, Spain; 2 Department of Cardiac Surgery, University Hospital Zürich, Zürich, Switzerland; 3 Department of Cardiothoracic Surgery, The University of the Free State, Bloemfontein, South Africa

**Keywords:** Hypertrophic cardiomyopathy, left ventricular outflow tract obstruction, septal myectomy, outcomes

A peak gradient ≥50 mmHg and advanced symptoms expressed as New York Heart Association (NYHA) classess III/IV, angina and/or syncope are acknowledged indications to surgically treat hypertrophic obstructive cardiomyopathy (HOCM). This is still a relatively neglected disease despite surgical myectomy has shown extreme efficacy since it was introduced by Morrow and Brockenbrough in 1961.^(1)^ Over the past six decades, credit went to Morrow and Brockenbrough, although Kirklin and Ellis also published their first experience with resection of diffuse subaortic stenosis through the left ventricle.^(2)^ There were only a couple of months difference in the chronology of these pioneering contributions and both groups reported surgery in critically symptomatic young patients. This is the gold standard sixty years later.

Over the years, terminology changed, adapting to evolving knowledge and evidence. Morrow^(1)^ and Kirklin^(2)^ discussed about diffuse hypertrophic subaortic stenosis as an independent condition. The word idiopathic was later replaced as cardiac sarcomere (or sarcomere-related) pathogenic genes were identified, with immediate impact on understanding the inheritance of a large proportion of hypertrophic cardiomyopathy (HCM) patients.^(3)^ The world of cardiomyopathies has seen extraordinary progress with entities like arrhythmogenic, restrictive or non-compacted^(4,5)^ and it is clear today likely that further disease-causing genetic triggers will be identified in HCM.^(6)^In less than a century the medical community has modulated the outcomes of a disease perceived with dismal prognosis into very often a normal life span. That has been possible with a myriad of tailored interventions that also include appropriate drug intervention, septal reduction therapies and the judicious use of defibrillators to prevent sudden death in selected HCM patients.

Despite all this progress, surgical septal myectomy (SM) continues to be the best way to treat patients with an obstructive physiology who have an indication for relief of the left ventricular outflow tract (LVOT) despite the introduction of alternative therapies like alcohol septal ablation (ASA) by Sigwart in 1995.^(7)^ The results of this surgical septal reduction are well documented in the literature and SM is supported in the American Heart Association/American College of Cardiology (AHA/ACC) most recent guidelines^(8)^ with a class of recommendation 1 with level of evidence B-NR (Non-randomized) due to the lack of controlled studies. The latest European Society of Cardiology (ESC) clinical practice guidelines from 2014,^(9)^ gave SM a more restricted role in the treatment of HOCM, stating “…Septal myectomy, rather than ASA, is recommended in patients with an indication for septal reduction therapy and other lesions requiring surgical intervention (e.g. mitral valve repair/replacement, papillary muscle intervention)…”, giving a class I recommendation and level of evidence C. This was expected due to the different and historically skewed management of HOCM in Europe, favoring ASA. These latest ESC guidelines were issued seven years ago. More data have been accumulated in the literature which confirm the excellent efficacy and reliability of SM across the entire spectrum of HOCM including all anatomical/phenotypical variants that cannot be addressed in any way by any pharmacological therapy or ASA.

The debate is still ongoing. Those favoring SM have the perspective of sixty years of surgery and, more importantly, accumulated data over time with very long periods of follow-up (reported up to 25 years after SM), still unreported by any other approach of septal reduction.^(10,11)^ The survival at 25 years of follow up after SM has been described by Schaff recently. The male population with a mean age of 53 years at the time of SM (who presented in a less advanced disease-stage than women) demonstrates an expected survival that matches the corresponding age-matched, sex-matched United States population. With those numbers in our hand the perception of a “curative” surgical intervention for HOCM gains propulsion. Undeniably, this is a call for improvement of care for women with HCM. Those in favour of ASA understand that the eventual indications are rather limited in comparison with SM.^(12)^ Pharmacological therapies remain the first line of therapy although the options are still limited to nonspecific medications, frequently offering suboptimal results.^(13)^ Myosin modulation with Macavamtem (Bristol Myers Squibb, New York, USA) is currently being investigated by the community, although the only Phase III trial only compared the investigational drug with placebo.^(14)^ This, at the moment, raises important doubts and questions as this trial has only confirmed that with Mavacamten, the persistence of obstruction was high in a less symptomatic population, improvement in pVO_2_ was suboptimal in a young study cohort (mean age 58 years), and the lifetime tolerance remains unassessed. Other aspects to be discussed include the risk of left ventricular dysfunction in the long-term and further side-effects.^(15)^ In any case, these preliminary results seem to be far away from the outcomes of SM across the spectrum of centers and authors. The role of the billionaire involvement of the pharmacological industry in developing this drug has to be also taken into account as it is likely that many trials will be organized in the future aiming at supporting its introduction in clinical practice, of course searching for the non-surgical Holy Grail. Of interest is that the Food and Drug Administration (FDA) accepted Bristol Myers Squibb's application for Mavacamten in symptomatic HOCM in March 2021.^(16)^ Regardless of the accumulated knowledge, experience and outcomes with SM over six decades, the controversy in practices worlwide is still out there. We addressed this not so long ago, aiming at honestly describing what is best for which patient.^(17)^ Avoiding pharmacological, percutaneous or surgical interventions while restoring normal quality of life and survival in HOCM is the ultimate will we all share. For the time being SM is the only proven one-time tailored intervention that may achieve such goals of care.

All of the above serves to introduce the readership of the *Asian Cardiovascular and Thoracic Annals* to this special issue which focus on Surgery for Hypertrophic Cardiomyopathy. *Asian Cardiovascular and Thoracic Annals* is dedicated to the Asian cardiovascular and thoracic community and aims at reaching all professionals involved in the field, offering the most updated aspects of cardiovascular and thoracic medicine and surgery. The discussions with the Editor-in-Chief and the Editorial Board led us to choose such a complex cardiac genetic disorder for a special issue of the Journal for 2021.

The goals of this special issue went beyond the purely surgical aspects of the disease. Although SM is out there effectively contributing to the patients’ benefit, there is much more to do. Creating awareness among patients, surgeons, cardiologists, general practitioners and allied professionals is a major goal; we expect that this special issue will seriously contribute to make the readership understand that SM is a fundamental part of the entire process of diagnostics and therapy for HOCM.

The variety of topics presented have been grouped in three major sections. The first is an overview of the disease from the perspective of the cardiologists who are mainly dedicated to the disease and understand that SM is the way to potentially cure a substantial proportion of the HOCM patients. Then, we incorporated the view of colleagues at the forefront of a leading HCM association aiming at providing support, advocacy and education for the benefit of the community. How to educate surgeons to start successful HOCM programs has been found of importance for those who are willing to treat the disease in an organized manner.

The second section is dedicated to preintervention and intraoperative imaging and management of the HOCM patient followed by the methodology to effectively assess the relief of LVOT obstruction. All together focusing on the key role of the dedicated anesthesiologists in the surgery for HOCM. Postoperative management is also described.

Finally, clinical experiences from all across Asia and the Pacific Rim will be described by different teams from different countries. The goal, as stated, is to distribute and exchange useful information to create awareness beyond those centers of excellence where HCM is diagnosed and treated. Some important concepts like discussing HCM multidisciplinary teams, reinforcing SM as the gold standard and the need for the highest quality pre-, intra- and postoperative management are presented herein, this bringing even more value to this special issue.

In summary, this special issue of the *Asian Cardiovascular and Thoracic Annals* intends that once awareness is created, dedicated multidisciplinary teams in Asia and the Pacific Rim will spread knowledge and that septal reduction therapies, in particular SM, the gold standard, become routine for the benefit of largest number of patients possible. Asia has the numbers and data generating knowledge have to follow.^(18)^

The Editor-in-Chief and the Guest Editors would also like to express our gratitude to all individual authors and centres graciously collaborating with *Asian Cardiovascular and Thoracic Annals*. We understand that all contributors stole precious time from their busy agendas to allow us constructing this Special Issue and this must be recognized. Having said that, it is also our duty to specifically recognize what the worldwide recognized as leaders in the field have done for the *Asian Cardiovascular and Thoracic Annals* and us. The cardiologists Drs. Barry J. Maron and Martin S. Maron are at the forefront of the development of knowledge on HCM worldwide for more than twenty years and they offered us the most valuable point of view of the clinician about this complex disease, setting the pace for a judicious, unbiased, practical and most honest approach for the benefit of the patient. Drs. Harztell Schaff and Joseph Dearani from the Mayo Clinic and Dr Nicholas Smedira from the Cleveland Clinic and their esteemed colleagues have collected separately the largest experiences on the surgical treatment of HOCM in the last three decades. They are internationally recognized by their scientific approach in surgery and their role as teachers and mentors has paved the way for a number of surgeons to understand, execute and disseminate surgical knowledge wherever new programmes have been established.

## References

[1] Morrow AG Brockenbrough EC . Surgical treatment of idiopathic hypertrophic subaortic stenosis: technic and hemodynamic results of subaortic ventriculomyotomy. Ann Surg 1961; 154: 181–189. 13772904 10.1097/00000658-196108000-00003 PMC1465878

[2] Kirklin JW Ellis FH . Surgical relief of diffuse subvalvular aortic stenosis. Circulation 1961; 24: 739–742. 14456314 10.1161/01.cir.24.4.739

[3] Geisterfer-Lowrance AA Kass S Tanigawa G , et al. A molecular basis for familial hypertrophic cardiomyopathy: a beta cardiac myosin heavy chain gene missense mutation. Cell 1990; 62: 999–1006. 1975517 10.1016/0092-8674(90)90274-i

[4] Olson TM Michels VV Thibodeau SN , et al. Actin mutations in dilated cardiomyopathy, a heritable form of heart failure. Science 1998; 280: 750–752. 9563954 10.1126/science.280.5364.750

[5] McKenna WJ Maron BJ Thiene G . Classification, epidemiology, and global burden of cardiomyopathies. Circ Res 2017; 121: 722–730. 28912179 10.1161/CIRCRESAHA.117.309711

[6] Maron BJ Maron MS Semsarian C . Genetics of hypertrophic cardiomyopathy after 20 years: clinical perspectives. J Am Coll Cardiol 2012; 60: 705–715. 22796258 10.1016/j.jacc.2012.02.068

[7] Sigwart U . Non-surgical myocardial reduction for hypertrophic obstructive cardiomyopathy. Lancet 1995; 346: 211–214. 7616800 10.1016/s0140-6736(95)91267-3

[8] Ommen SR Mittal S Burke MA , et al. AHA/ACC guideline for the diagnosis and treatment of patients With hypertrophic cardiomyopathy: executive summary: a report of the American college of cardiology/American heart association joint committee on clinical practice guidelines. J Am Coll Cardiol 2020; 2020: 3022–3055. 10.1016/j.jacc.2020.08.044 33229115

[9] Elliott PM Anastasakis A Borger MA , et al. ESC Guidelines on diagnosis and management of hypertrophic cardiomyopathy: the task force for the diagnosis and management of hypertrophic cardiomyopathy of the European Society of Cardiology (ESC). Eur Heart J 2014; 2014: 2733–2779. 10.1093/eurheartj/ehu284 25173338

[10] Nguyen A Schaff HV Ommen SR , et al. Late health status of patients undergoing myectomy for obstructive hypertrophic cardiomyopathy. Ann Thorac Surg 2021; 111: 1867–1875. 33127406 10.1016/j.athoracsur.2020.09.011

[11] Alashi A Smedira NG Hodges K , et al. Outcomes in guideline-based class I indication versus earlier referral for surgical myectomy in hypertrophic obstructive cardiomyopathy. J Am Heart Assoc 2021; 10: e016210. 33342243 10.1161/JAHA.120.016210 PMC7955478

[12] Veselka J Jensen MK Liebregts M , et al. Long-term clinical outcome after alcohol septal ablation for obstructive 2021 hypertrophic cardiomyopathy: results from the euro-ASA registry. Eur Heart J 2016; 37: 1517–1523. 26746632 10.1093/eurheartj/ehv693

[13] Stătescu C Enachi Ș Ureche C , et al. Pushing the limits of medical management in HCM: a review of current pharmacological therapy options. Int J Mol Sci 2021; 22: 7218. 10.3390/ijms22137218 PMC8268685 34281272

[14] Olivotto I, Oreziak A, Barriales-Villa R, et al. EXPLORER-HCM study investigators. Mavacamten for treatment of symptomatic obstructive hypertrophic cardiomyopathy (EXPLORER-HCM): a randomised, double-blind, placebo-controlled, phase 3 trial. Lancet 2020; 396: 759–769. 32871100 10.1016/S0140-6736(20)31792-X

[15] Quintana E Bajona P Myers PO . Mavacamten for hypertrophic obstructive cardiomyopathy. Lancet 2021; 397: 369. 10.1016/S0140-6736(20)32384-9 33516331

[16] https://news.bms.com/news/corporate-financial/2021/U.S.-Food-and-Drug-Administration-FDA-Accepts-Bristol-Myers-Squibbs-Application-for-Mavacamten-in-Symptomatic-Obstructive-Hypertrophic-Cardiomyopathy-oHCM/.

[17] Mestres CA Bartel T Sorgente A , et al. Hypertrophic obstructive cardiomyopathy: what, when, why, for whom? Eur J Cardiothorac Surg 2018; 53: 700–707. 29438530 10.1093/ejcts/ezy020

[18] Wan S . Mitral valve surgery at the oriental crossroad. Asian Cardiovasc Thorac Ann 2020; 28: 357–359. 33023306 10.1177/0218492320957133

